# Pilot field testing of the chronic pain classification for ICD-11: the results of ecological coding

**DOI:** 10.1186/s12889-018-6135-9

**Published:** 2018-11-07

**Authors:** Antonia Barke, Beatrice Korwisi, Hans-Raimund Casser, Egil A. Fors, Christian Geber, Stephan A. Schug, Audun Stubhaug, Takahiro Ushida, Thomas Wetterling, Winfried Rief, Rolf-Detlef Treede

**Affiliations:** 1 Department of Psychology, Division of Clinical Psychology and Psychotherapy, Philipps University Marburg, Gutenbergstr. 18, 35032 Marburg, Germany; 2 DRK Schmerz-Zentrum, Auf der Steig 16, 55131 Mainz, Germany; 3 General Practice Research Unit, Norwegian University of Science and Technology, NO-7491 Trondheim, Norway; 4 University of Western Australia & Royal Perth Hospital, Perth, WA 6847 Australia; 5 Oslo University Hospital, University of Oslo, Kirkeveien 166, None, 0450 Oslo, Norway; 6 Aichi Medical University, Nagakute, Aichi 480-1195 Japan; 7 Department of Neurophysiology, Centre for Biomedicine and Medical Technology Mannheim, Medical Faculty Mannheim, Heidelberg University, Ludolf-Krehl-Str. 13–17, 68167 Mannheim, Germany

**Keywords:** Field testing, Chronic pain, Classification, Clinical utility, Diagnostic categories, Ecological coding, ICD-11

## Abstract

**Background:**

A task force of the International Association for the Study of Pain (IASP) has developed a classification of chronic pain for the ICD-11 consisting of seven major categories. The objective was to test whether the proposed categories were exhaustive and mutually exclusive. In addition, the perceived utility of the diagnoses and the raters’ subjective diagnostic certainty were to be assessed.

**Methods:**

Five independent pain centers in three continents coded 507 consecutive patients. The raters received the definitions for the main diagnostic categories of the proposed classification and were asked to allocate diagnostic categories to each patient. In addition, they were asked to indicate how useful they judged the diagnosis to be from 0 (not at all) to 3 (completely) and how confident they were in their category allocation.

**Results:**

The two largest groups of patients were coded as either chronic primary pain or chronic secondary musculoskeletal pain. Of the 507 patients coded, 3.0% had chronic pain not fitting any of the proposed categories (97% exhaustiveness), 20.1% received more than one diagnosis. After adjusting for double coding due to technical reasons, 2.0% of cases remained (98% uniqueness). The mean perceived utility was 1.9 ± 1.0, the mean diagnostic confidence was 2.0 ± 1.0.

**Conclusions:**

The categories proved exhaustive with few cases being classified as unspecified chronic pain, and they showed themselves to be mutually exclusive. The categories were regarded as useful with particularly high ratings for the newly introduced categories (chronic cancer-related pain among others). The confidence in allocating the diagnoses was good although no training regarding the ICD-11 categories had been possible at this stage of the development.

## Background

The International Association for the Study of Pain (IASP) defines pain as an unpleasant sensory or emotional experience associated with actual or potential tissue damage or described in terms of such damage [1]. Chronic pain is pain that persists or recurs for longer than three months [2, 3]. Chronic pain affects more than 20% of the population worldwide [4–7], accounts for up to 20% of physician visits [8, 9], and emerged as an important component in the global burden of disability [10]. However, despite their importance, in the current version of the *International Classification of Diseases* (ICD-10), chronic pain conditions are not recognized in a systematic way. Adequate representation in the ICD has far-reaching consequences. The ICD is the global standard of diagnostic classification and serves a wide range of purposes: by international treaty, it supports the world-wide collection of health statistics and the collected data provide the basis most governments use for their health policy, planning, and resource allocation [11]. In addition, the codified criteria identify the conditions for research, so the ICD diagnoses inform research agendas. Lastly, adequate diagnoses are essential for treatment choices [12, 13].

Responding to this need, the IASP established a task force consisting of pain experts from around the world to develop a pragmatic classification of chronic pain for the inclusion into the eleventh revision of the ICD. The task force has presented a pragmatic, research-based classification proposal, which comprises seven categories of chronic pain conditions [2, 3]:

1. Chronic primary pain (e.g. irritable bowel syndrome, ‘non-specific’ chronic low back pain, fibromyalgia) [14]
2. Chronic cancer-related pain (e.g. chronic cancer pain, chronic post-chemotherapy pain) [15]
3. Chronic postsurgical and posttraumatic pain (e.g. chronic pain after amputation, chronic pain after burns injury) [16]
4. Chronic neuropathic pain (e.g. chronic painful polyneuropathy, chronic central post-stroke pain) [17]
5. Chronic secondary headache or orofacial pain (e.g. chronic orofacial muscle pain) [18]
6. Chronic secondary visceral pain (e.g. chronic visceral pain from persistent inflammation or from vascular mechanisms) [19]
7. Chronic secondary musculoskeletal pain (e.g. chronic musculoskeletal pain from persistent inflammation, chronic musculoskeletal pain associated with osteoarthritis) [20].

Importantly, several of these chronic pain conditions hitherto have not been represented in the ICD, e.g. chronic primary pain, chronic neuropathic pain and chronic cancer-related pain. The seven large classes of chronic pain comprise more detailed subdiagnoses, capturing well-described pain problems (see examples in parentheses). A patient presenting with chronic back pain, for example, should be screened for red flags related to chronic secondary musculoskeletal back pain [20]; if there are no red flags, he or she would receive the diagnosis of chronic primary musculoskeletal pain, which is a more detailed subcategory of chronic primary pain [14]. This contrasts with the situation in ICD-10, in which there are a great number of codes for chronic back pain, many of them widely criticized [14, 20, 21]. In addition, the only diagnoses represented are ones that are regarded as chronic secondary musculoskeletal pain by the new classification. All chronic pain diagnoses can be combined with optional specifiers encoding pain severity (which includes pain intensity, pain-related disability, and pain-related emotional distress), the temporal course of the pain, and the presence of psychosocial factors [2, 3].

With the ICD-11, the WHO has introduced several new features [22]. Among others, the *World Health Organisation* (WHO) implemented multiple parenting. Multiple parenting means that diagnostic entities can have more than one so-called parent, i.e. be listed under more than one heading. This overcomes the problem that, previously, one and the same disease could be classified, e.g. according to etiology (as a neoplasm) and according to site (genito-urinary system) with different codes. Now, the different places will all link to one and the same code. In the area of pain, an entity such as “Chronic chemotherapy-induced pain” can now be subordinate to, e.g. “Chronic cancer pain” (etiology) and “Chronic neuropathic pain” (mechanism). By allowing the same entity to be included in two or more categories, multiple parenting represents an advance in the systematic structure [2] that often also corresponds to different medical specialties (i.e. oncologist and neurologist).

Please note that the present authors use the term ‘diagnosis’ in a technical sense. The WHO recognizes diseases, disorders, signs, symptoms, injuries, and reasons for encounter as entities in the foundation of ICD-11. We use the term diagnosis for the allocation of a code for a cluster of frequently co-occurring symptoms, for which further inclusion and exclusion criteria apply. This may be a disease or a disorder. We do not intend to imply any further claims regarding a possible etiology or disease entity by calling something ‘a diagnosis’.

Any new classification must demonstrate its quality [11, 13]. Three relevant dimensions determining a classification’s quality are reliability, validity, and utility. Reliability refers to the question of whether the same entity is coded in the same way on different occasions, either by different raters (inter-rater reliability) or by the same rater (intra-rater reliability). Several factors impact reliability measures: on the raters’ side, reliability may be improved by training and conscientious practice; on the classification’s side, clear, operationalized criteria and limited complexity facilitate reliable classification [23, 24]. Validity has been discussed with reference to particular diagnoses and whole classification systems [25, 26]. Applied to a classification as a whole, a valid classification would be one where the boundaries of the diagnostic categories defined in the conceptual realm correspond to separate entities in external reality. It is apparent that this is an ideal, attainable only in part and difficult to test empirically. With respect to pain classification, the difficulty is exacerbated since pain is a subjective experience and external references are lacking [27]. Clinical use, alongside public health use, is one of the main purposes of the ICD [28], and the (WHO) has prioritized improvement of clinical utility during the ICD revision [11, 26, 28]. Clinical utility is defined as the degree to which a classification system conceptualizes diagnostic entities, contributes to the selection of adequate treatment interventions, predicts clinical management needs, is applicable in clinical practice [29], provides information about prognosis and treatment outcome [26], and facilitates communication about the phenomena described. Moreover, a clinically useful classification system is easy to use [11]. Reliability as well as clinical utility is improved if the categories of a classification system have clear boundaries [13] and the categories are mutually exclusive, exhaustive, reliable, biologically plausible, and simple [30].

Two types of field tests can be distinguished to evaluate clinical utility [11]. Formative field tests are implemented during the process of developing a new classification in order to obtain information on how to improve the structure and content of the classification system. Evaluative field tests are administered once a first draft of the new classification system has been finalized, to evaluate different aspects of its application [29]. The field test described here represents a formative field test. This implies that the classification was tested at the implementation level it had at the time of testing, which meant that only the seven top level diagnoses could be tested. The fact that only the top level diagnoses could be tested had important consequences: One of the consequences for the testing presented here was that due to multiple parenting some diagnoses are correctly subsumed under more than one heading. In the final version (available at the website of the ICD-11: https://icd.who.int/dev11), this will be solved on the next level of the classification and does not constitute a problem with a category boundary.

The goal of the present pilot field test was the assessment of:

1. the exhaustiveness of the basic categories of the proposed classification for chronic pain: how many cases of chronic pain cannot be classified by the proposed classification and would be relegated to the category “Chronic pain, unspecified”?
2. the clarity of the category boundaries: how many cases of chronic pain would be allocated to more than one category?
3. the perceived usefulness of the categories;
4. the confidence with which the coding clinicians chose each category.

In order to gather pilot data with regard to these questions, clinicians from several pain centers and one primary care center participated and classified a number of consecutive patients on the basis of the proposed classification.

## Methods

### Participating institutions and raters

Several institutions on three continents participated in the preliminary testing, which was carried out from 15 July – 31 August 2016 upon request by the WHO. The participating raters worked at tertiary pain centers, except for one Norwegian primary care center. (See Table 1 for an overview of the participating centers). The choice of participating centers was mainly guided by their ability to carry out the testing in the timeframe of the ICD-11 preparation process, both in terms of resources and numbers of patients with chronic pain seen by them. Centers were selected from IASP collaboration centers. The raters were clinicians experienced with regard to chronic pain, but without any formal training with respect to the ICD-11, because such training was unavailable at that stage in the development. The definitions themselves and the overview article [2] were the only aid. Raters allocated ICD-11 codes to consecutive patients as they were seen, and at conclusion of the data collection time had coded a total number of 507 patients.

**Table 1:** Participating institutions, cases coded by each center and the number of raters

Institution	Country	Type	Patients rated	Number of raters
Several Multidisciplinary Pain Centers (Aichi Med. Univ., Ehime Univ., Jikei Med. Univ., Kyushyu Univ., Univ. Tokyo, Osaka Univ., Saga Univ., Toyama Univ., Nihon Univ., Fukushima Med. Univ., Juntendo Univ.)	Japan	Tertiary pain centers	91	14
Pain Medicine Center Royal Perth Hospital	Australia	Tertiary pain center	62	4
Deutsches Rotes Kreuz Schmerzzentrum (Mainz)	Germany	Tertiary pain center	150	2
Oslo University Hospital, Dept. of Pain Management and Research	Norway	Secondary and tertiary care	103	4
Edda Medical Center/Leangen Medical Center/Heimdal Helsehus Medical Center (Trondheim)	Norway	Primary care	101	3

### Material

All institutions received the definitions as they are provided in the ICD-11 and operationalized diagnostic criteria for the following seven diagnoses:

1. Chronic primary pain
2. Chronic cancer-related pain
3. Chronic postsurgical and posttraumatic pain
4. Chronic neuropathic pain
5. Chronic secondary headache or orofacial pain
6. Chronic secondary visceral pain
7. Chronic secondary musculoskeletal pain

These diagnoses represent the top level of the classification. Including the individual diagnostic categories below the top level was not possible because they had not been implemented in the ICD-11 foundation layer at the time.

### Procedure

The participating institutions were asked to code 100–150 consecutive patients according to the criteria provided and report on the diagnoses, whether the raters thought the patients’ conditions would fall under more than one category (to assess category boundaries) or whether they did not fit any of the categories (to assess the exhaustiveness of the categories combined). In addition, the raters were asked to rate the utility of each diagnosis and subjective diagnostic certainty with which they allocated it.

### Diagnostic code

For each coded patient, it was recorded which diagnoses he or she had received routinely on the basis of ICD-10 and the diagnoses he or she would receive according to the ICD-11 diagnostic categories (1–7). For primary care, patients also had received pain diagnoses from the International Classification of Primary Care, Second Edition (ICPC-2).

### Category exhaustiveness

If the diagnosis did not fit any of the ICD-11 categories, the raters were instructed to record this fact. For this purpose, it was asked: “Did the patient have chronic pain that did not fit any of the categories?” The answers were used to investigate the proportion of diagnoses the seven categories in combination could classify. A high number of affirmative answers to this question would mean that many instances of chronic pain cannot be classified with the proposed system; conversely, low numbers of affirmative answers point to good coverage of the field of chronic pain by the proposed classifications.

### Category boundaries

In order to determine the clarity of the category boundaries, the raters were asked whether the pain the patient reported fitted into more than one category and, if so, into which categories. Affirmative answers to this question were taken to indicate a possible problem with category boundaries with a few provisos. Positive answers to this question may have different causes. Firstly, the patient could have two comorbid pain complaints (i.e., chronic tension-type headache and knee osteoarthritis) and would naturally receive two diagnoses. These cases were easily discerned on the basis of the ICD-10 codes, and were counted as co-existing pain conditions rather than double categories. Secondly, the patient could have a pain disorder that would be an instance of double parenting, such as cancer pain of a neuropathic nature. In the actual classification, this would receive a single diagnostic code that will be accessible via cancer-related pain and via neuropathic pain. Because the present field testing only extended to a level at which the double parenting could not be expressed, double category assignments of this nature were regarded as artifacts of the testing procedure. The remaining double codings were deemed to be due to a lack of clarity of the category boundaries. These cases were counted and high proportions of these cases could indicate a possible problem with category boundaries or their perception by the raters.

### Perceived clinical utility and subjective diagnostic certainty

In order to assess perceived clinical utility each clinician rated on a four-point scale from 0 (*not at all*), 1 (*somewhat*), 2 (*very*) to 3 (*completely*) how useful they felt the diagnosis was for each patient. The subjective feeling of diagnostic certainty was rated on a four-point scale from 0 (*not at all confident*), 1 (*somewhat*), 2 (*very*) to 3 (*completely confident*). Mean scores for perceived utility and diagnostic certainty were calculated for each category, with higher scores indicating higher utility and confidence, respectively.

### Data analysis

The data were analyzed by descriptive methods. We counted the diagnoses that the patients received using the old ICD-10 classification and calculated the average number per patient. For the new classification for ICD-11, we also counted how many diagnostic codes each patient had received. The maximum here would be 7 (all diagnoses applied to the person) and the minimum 0 (none of the 7 categories applied). To establish category boundaries, theoretically, each patient should fall into only one of the seven categories. Cases in which the number of diagnoses allocated was higher than 1 were therefore taken as indicative of a possible problem with category boundaries, provided, they were not occasioned by one of the possibilities outlined above in Section "Category Boundaries". Cases in which the number was 0, were counted and assessed separately as cases in which no diagnosis could be assigned by the raters: this represents the case of chronic pain that cannot be classified within the system. In order to be certain that it had not just been an oversight, we counted the answers to the explicit question of whether the patient had chronic pain that was unclassifiable by the categories provided and calculated the percentage of all cases that remained unclassifiable within the tested classification. A high percentage of unclassifiable cases would be indicative of a problem with the exhaustiveness of the categories. For each category, means and standard deviations for the raters’ subjective diagnostic confidence and utility judgements are reported.

## Results

### Diagnostic codes

**Fig. 1:**
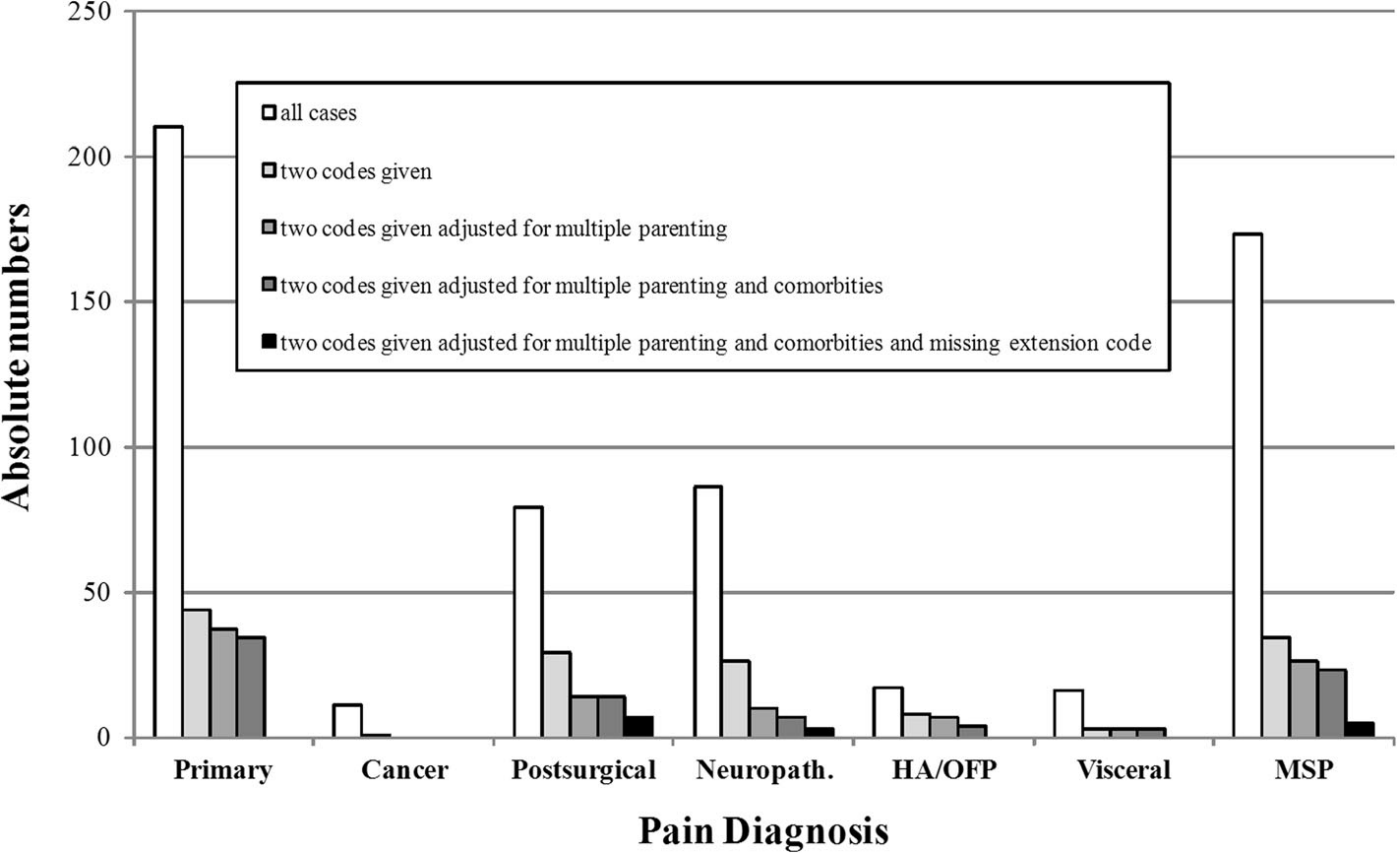
All cases (absolute numbers) and cases in which more than one diagnostic code was assigned, adjusted for various coding artefacts, such as true comorbidity, multiple parenting, and missing extension codes. HA/OFP: Chronic headache or orofacial pain, MSP: Chronic secondary musculoskeletal pain

The most frequently given codes were chronic primary pain and chronic secondary musculoskeletal pain, probably partly reflecting their high incidence in primary, secondary, and tertiary care. However, the low frequency of chronic cancer-related pain, chronic headache or orofacial pain and chronic secondary visceral pain may also reflect a recruitment bias in this small group of participating centers. The patients received on average 1.2 ± 0.7 ICD-11 codes (range 1–3). (See Fig. 1 for the distribution across the seven ICD-11 categories). The patients received, on average, 1.7 ± 1.2 ICD-10 codes (range: 1–7). The most frequent ICD-10 codes were: F45.4 persistent somatoform pain disorder (*n* = 60), M54.4 lumbago with sciatica (*n* = 48), and M54.5 low back pain (*n* = 43); the most frequent ICPC-2 codes were L18 muscle pain (*n* = 13), A01 Pain general/multiple sites (*n* = 9), L02 Back symptom/complain (*n* = 8). In seven cases (1.2%), the rater used a clearly erroneous ICD-11 code (as compared with the ICD-10 codes and comments provided), e.g. coding a knee problem as visceral pain.

### Exhaustiveness

Of the 507 coded patients, 15 were reported to have chronic pain that failed to be represented by any of the seven categories. These constitute 3.0% of the total and, following ICD-11 procedure, would be classified as “Chronic pain, unspecified”.

### Category boundaries

Of the 507 patients coded, 102 patients (20.1%) were classified as belonging to more than one category. Of these, 36 (7.1%) were due to co-existence of two separate pain conditions, and 23 (4.5%) to the fact that the level of testing could not take into account multiple parenting. In 33 (6.5%) cases, it appears that chronic primary pain was given as an additional comorbid diagnosis in order to express the presence of psychosocial factors influencing a diagnosis of secondary pain. These instances will later be solved by use of an extension code for psychosocial factors with all chronic pain diagnoses, and hence will not constitute a problem with category boundaries. After removal of all three types of artificial cases, only 10 cases remained as potential double classifications (2.0%) (see Fig. 1).

### Perceived clinical utility and confidence

Generally, the diagnoses were rated as very useful (mean: 1.9 ± SD: 1.0; theoretical range: 0–3). The categories judged as most useful were “Chronic cancer-related pain” (2.7 ± 0.5) and “Chronic neuropathic pain” (2.3 ± 0.8). The raters were confident in applying the diagnoses (2.0 ± 1.0). The highest confidence was expressed with regard to chronic cancer-related pain (2.8 ± 0.4) and chronic neuropathic pain (2.2 ± 0.8). In general, the divergence between the diagnostic categories was small (see Table 2).

**Table 2:** Mean ratings of usefulness for the proposed categories and subjective confidence in allocating the cases to the respective categories

Diagnostic entity	Perceived utility (mean ± SD)	Subjective confidence (mean ± SD)
Chronic primary pain	1.7 ± 1.0	1.8 ± 1.0
Chronic cancer-related pain	2.7 ± 0.5	2.8 ± 0.4
Chronic postsurgical and posttraumatic pain	2.0 ± 0.9	2.2 ± 0.9
Chronic neuropathic pain	2.2 ± 0.8	2.2 ± 0.8
Chronic secondary headache or orofacial pain	1.9 ± 0.7	2.0 ± 0.6
Chronic secondary visceral pain	1.8 ± 0.8	1.8 ± 0.9
Chronic secondary musculoskeletal pain	1.9 ± 0.9	2.0 ± 0.9

Theoretical range of the ratings: 0 (not at all) to 3 (completely)

## Discussion

The present study is the first to present field testing data regarding the proposed classification of chronic pain for the ICD-11. In a naturalistic setting, clinicians in four countries applied the ICD-11 classification to consecutive patients testing the feasibility of its application. They provided ratings of the clinical utility of the suggested categories and their subjective diagnostic confidence. We explored whether the combined categories exhausted the encountered pain conditions and whether overlapping category assignments pointed to a problem with category boundaries.

The clinicians involved in the testing were (except in Australia) wholly independent of the task force that had developed the classification. The general feedback received from the participating institutions was very favorable, emphasizing the usability of, and need for, such a classification. The individual diagnoses proposed were, generally, judged useful. This was particularly true for diagnoses not hitherto included in the ICD, namely “Chronic cancer-related pain”, “Chronic neuropathic pain”, and “Chronic postsurgical and posttraumatic pain”. It is likely the utility will increase further, once sub-level diagnoses are included, such as chronic post-radiotherapy pain or chronic post-stroke pain. All participating clinicians were experienced in ICD-10 (ICPC-2, respectively) coding and familiar with pain syndromes, but specific training with regard to the proposed ICD-11 codes was not available at that stage of development of the classification. The fact that they still expressed good confidence indicates that the proposed system is readily usable. It is to be expected that – with training and practice – the reliability and subjective confidence will increase even further.

The proposal aimed at a good coverage of chronic pain syndromes. In the present field test, about 3% of cases were deemed not classifiable by the proposed classification and would have to be classed as “Chronic pain, unspecified”. It would be instructive to compare the data obtained by use of the ICD-11 with those in previous studies using the ICD-10. However, as chronic pain was not represented systematically in previous versions, epidemiological studies on chronic pain were not able to rely on ICD-10 codes [31–34], rendering it impossible to determine the percentage of patients who previously fell into the residual categories in the ICD-10. Our result indicates that the proposed ICD-11 categories capture a large majority of encountered chronic pain diagnoses. This is an excellent result since relegating conditions to a residual category is associated with a lack of information about treatment choice for the individual patient [35]. Scientifically, residual categories are associated with a shortage of research [36].

Unclear category boundaries limit the reliability of diagnostic categories. We therefore investigated the number of cases in which more than one category was assigned to a patient. Only in 20.1% of cases, two diagnoses were allocated. This number decreased substantially when true comorbidities were taken into account, and it was adjusted for cases that were due to the artificial situation that only the top level was coded where multiple parenting remained invisible. It is expected that the provision of second level diagnoses and their operationalized criteria in subsequent field trials will eliminate this problem. Excluding these cases, only 11.6% were left. A third type of case was observed in connection with chronic primary pain: a secondary pain syndrome with a clear underlying disease, e.g. chronic osteoarthritis, was exacerbated by psychological factors, e.g. anxiety. Sometimes, this seems to have led raters to conclude that the patient should also receive a diagnosis of chronic primary pain. This is not correct and the problem will be addressed once the specifiers are included in the field testing (which was not an option at this time). Since the specifier “with psychosocial factors” can be combined with all diagnoses in the classification, it will be easy to represent such cases. If the numbers are adjusted for all these cases, only 2.0% of cases remain in which two diagnoses were allocated for a single condition. This points to a very good demarcation between the categories.

In addition, we had asked the raters to judge the perceived utility of the diagnosis for each individual case and all diagnoses were rated as very useful, with the highest scores for chronic cancer-related pain, chronic neuropathic pain and chronic postsurgical and posttraumatic pain. However, the utility to be expected from the implementation of the new diagnoses could not be fully represented in the ratings of the clinicians for individual cases. The extremely positive ratings for categories like cancer and neuropathic pain could be additionally motivated by preferences of some raters (and health care systems), especially in tertiary care, for pathology-rooted categories. Considering the presently unsatisfactory situation with regard to (what will in future be called) “primary pain conditions”, the relative improvement in utility for the classification of primary pain could be much higher than captured here.

It should be noted that the reported numbers of the encountered diagnoses do not indicate prevalence of the conditions. Sampling consecutive cases after a start date does not allow for any such conclusion; it only shows what kinds of conditions were encountered in the participating pain centers during that time. Still, the newly introduced “Chronic primary pain” diagnosis was the most frequently used single diagnosis. These first results indicate that this new diagnosis is well accepted, and a major improvement compared to existing concepts (e.g., somatoform pain disorder; functional pain syndromes). We had also asked for ICD-10 codes and ICPC-2 codes for the patients, and inspection of these codes showed that many patients had three and more ICD-10 codes. Although no definite conclusion can be drawn due to the different levels of the coding (top level in the field testing vs. individual codes), it was also apparent that with the new classification, for a majority of these cases, fewer, and more suitable, diagnoses would become available.

### Recommendations

Individual comments received on cases and formulations were reviewed carefully by the task force and should be used to improve details of the classifications further. When implementing the classification, the relevant subcategories should be added. Formal training in the use of the classification should be available after its introduction into routine practice. Future research should address questions of inter-rater reliability and validity in different settings.

### Limitations and future directions

The field testing was part of a formative field test and helped to gain first insight into the clinical utility of the classification in routine practice prior to its implementation in the ICD-11. To be of relevance for the ICD-11 preparation process, the formative nature of the testing also meant that it had to be completed within a short time frame. Only this way would have allowed changes to main categories if a problem had been revealed by the testing. The main aim was to investigate *mutual category exclusiveness* and *joint exhaustiveness* rather than the smooth applicability in various settings. For this purpose, centers were chosen from the list of IASP cooperation centers, if enough patients with a variety of chronic pain complaints were seen and resources allowed clinicians to engage in double coding, i.e. accomplish the double coding in addition to discharging their clinical duties. These conditions were typically met by tertiary centers, but we are happy that one primary center participated. This represents a trade-off between speed of data collection/variety of patients over the investigation of a variety of settings. The relevance of the new pain classification in primary care centers is discussed elsewhere [21].

Further limitations were its pilot character, which implied the use of the classification’s top-level structure only, the lack of formal inclusion and exclusion criteria for the patients or in-depth data regarding their conditions. Moreover, no checks were implemented to assure that indeed consecutive patients were recruited, and that patients were not selectively excluded from this list. All raters were physicians who worked with patients with chronic pain and were experienced ICD-10 users, but had not received formal training using ICD-11 classifications. Different numbers of raters coded different patients of differing populations. This allowed the main purpose of the study (testing category boundaries and exhaustiveness) but made further analyses impossible. These analyses will be the focus of future field testing of the new classification that will implement various field testing strategies as e.g. elaborated by Keeley and colleagues [37]. Since it will no longer be a study of the formative phase, it will be able to use a full version of the classification as it has now been implemented in the ICD-11 and address questions of reliability and clinical utility.

This article was concerned mainly with clinicians allocating diagnoses on the basis of patient examinations. A further group of persons working with the ICD codes are non-clinicians coding diseases and health complaints from documentation for statistical or reimbursement purposes. Testing how the new classification of chronic pain performs in this context was beyond the scope of the present study, but will be part of a large WHO-led study regarding technical aspects of the ICD-11 codes, coding rules, the browser infrastructure and its search options as well as coding tools.

## Conclusion

This pilot field testing indicated that the proposed classification of chronic pain into one group of chronic primary pain syndromes and six groups of chronic secondary pain syndromes performed promisingly in real life, both in specialized pain centers and in one primary care center. The categories were exhaustive with very few cases being relegated to residual categories. They were also mutually exclusive and the raters were able to allocate single categories to the majority of cases even without any formal training. All proposed categories were regarded as clinically useful by the raters with a particular emphasis on the newly introduced categories of chronic cancer-related pain, chronic neuropathic pain, and chronic postsurgical and posttraumatic pain. Finally, the acceptance of the classification, and the subjective confidence in allocating the diagnoses was good and one of the participating sites continues to use the ICD-11 codes in their clinical practice.

## Abbreviations

CRPS: Complex regional pain syndrome
IASP: International Association for the Study of Pain
ICD: International Classification of Diseases
ICPC: International Classification of Primary Care
WHO: World Health Organisation
